# *Xestospongia muta* Fraction-7 and Linoleic Acid: Effects on *SR-BI* Gene Expression and HDL Cholesterol Uptake

**DOI:** 10.3390/md20120762

**Published:** 2022-12-04

**Authors:** Nurul Adila Azemi, Ahmad Khusairi Azemi, Luqman Abu-Bakar, Vigneswari Sevakumaran, Tengku Sifzizul Tengku Muhammad, Noraznawati Ismail

**Affiliations:** 1 Institute of Marine Biotechnology, Universiti Malaysia Terengganu, Kuala Terengganu 21030, Terengganu, Malaysia; 2 Faculty of Veterinary Medicine, Universiti Malaysia Kelantan, Kota Bharu 16100, Kelantan, Malaysia

**Keywords:** *Xestospongia muta*, linoleic acid, *SR-BI*, hypercholesterolemia, HDL

## Abstract

*Xestospongia muta* is a marine sponge belonging to the family Petrosiidae. It is an important source of biologically active marine natural products, with different kinds of essential fatty acids. Scavenger receptor class B type I (*SR-BI*) is the main receptor for high-density lipoprotein (HDL) cholesterol, which plays a pivotal role in preventing atherosclerosis. It removes cholesterol from HDL cholesterol, returning lipid-poor lipoprotein into blood circulation. The present study investigated the effects of *X. muta* Fraction-7 and linoleic acid on *SR-BI* gene expression and HDL cholesterol uptake. In vitro studies of the activity of *X. muta* and linoleic acid against the therapeutic target for hypercholesterolemia were conducted using the HDL receptor *SR-BI* via luciferase assay and HepG2 cells. In the present study, Fraction-7 of *X. muta* showed the highest expression level of the *SR-BI* gene via luciferase assay. Profiling of Fraction-7 of *X. muta* by GC-MS revealed 58 compounds, comprising various fatty acids, particularly linoleic acid. The in vitro study in HepG2 cells showed that the Fraction-7 of *X. muta* and linoleic acid (an active compound in *X. muta*) increased *SR-BI* mRNA expression by 129% and 85%, respectively, compared to the negative control. Linoleic acid increased HDL uptake by 3.21-fold compared to the negative control. Thus, the Fraction-7 of *X. muta* and linoleic acid have the potential to be explored as adjuncts in the treatment of hypercholesterolemia to prevent or reduce the severity of atherosclerosis development.

## 1. Introduction

Atherosclerosis is one of the lipid metabolic disorders and chronic inflammatory diseases, and occurs within the large- and/or medium-sized elastic vessels and intima or under the intima of muscular arteries. It is characterized by progressive lipid deposition, accompanied by fibrous tissue hyperplasia and inflammatory cell infiltration, forming atherosclerosis lesions or plaques, and eventually causing acute cardiovascular diseases from the rupture of plaques [1,2,3,4,5,6,7]. High-density lipoprotein (HDL) cholesterol is an independent risk factor for cardiovascular diseases. HDL is a part of the diagnostic criteria for metabolic syndrome and an important target for treating and preventing cardiovascular diseases [8,9,10]. The main form of anti-atherosclerosis function by HDL is through reverse cholesterol transport (RCT). RCT is an important part of cholesterol and lipoprotein metabolism, and this process involves the transport of excessive cholesterol from peripheral tissues to the liver for recirculation or excretion, which can reduce lipid deposition on the blood vessel wall [8,9,10,11].

Human scavenger receptor class B type I (*SR-BI*) is an integral membrane glycoprotein of the scavenger receptor family. It is mainly expressed in the liver and gastrointestinal tract, or is detected in macrophages and endothelial cells [8,9,10,12]. In the liver, *SR-BI* binds with HDL to mediate the selective uptake of HDL cholesterol with high affinity and high saturation, and this process is the final rate-limiting step of RCT. Therefore, the role of *SR-BI* is crucial in allowing HDL to remain functional, and transporting the plasma cholesterol away from blood vessels to the liver to be metabolized [13]. The overexpression of hepatocyte *SR-BI* promotes the reverse transport of HDL cholesterol, increases the HDL clearance rate in plasma, and reduces the atherosclerosis risk [8,9,10]. Therefore, *SR-BI* provides a new target for conventional drug treatments for atherosclerosis and hypercholesterolemia. Interest in the discovery of new chemicals or drugs is greater than ever due to the emergence of new diseases, the increasing number of drug-resistant diseases and pathogens, and, more importantly, the problems of finding new replacements and alternatives to current drugs that have adverse side effects on human health [13]. Marine natural products are a major source in the search for new drugs, especially marine sponges, due to their advantages such as low toxicity, abundance, low cost, and lesser side effects if used at the right dose.

According to the Natural Product Report, about 300 novel compounds were determined from sponges in the decade 2001–2010 and estimated in the decade 2011–2020 on average each year. In addition, novel compounds from sponges have increased even more [14,15,16]. The chemical structure of the compounds from sponges is also diverse, such as fatty acids, glycolipids, macrolide, peptides, peroxide, polyketide, quinone, steroids, terpenoids, and alkaloids. Their fatty acids and ester derivatives exhibit anti-inflammatory activity, quinone derivatives are characterized by anti-malarial and anti-HIV properties, polyketides, polybrominated peptides, and macrolactam have anti-microbial properties, and dihydropyridine alkaloids have a protective effect on nerve cells [14,17]. *Xestospongia muta* (Schmidt, 1870; family Petrosiidae), commonly called the giant barrel sponge, is the largest species of sponge found growing in the Caribbean coral reef. It provides a substrate for numerous organisms, is involved in marine nutrient dynamics, and is a key source of pharmaceutical compounds [18,19,20].

Studies have reported that compounds extracted from *X. muta* showed anti-microbial [21,22,23], anti-tumor [24], anti-malarial [25], and anti-HIV [26] properties. Previously, *X. muta* has been considered an important source of fatty acids, including 9–12 octadecadienoic methyl ester/linoleic acid (LA, polyunsaturated omega-6 fatty acid) [27]. It has been reported that a diet rich in LA leads to a reduction in cholesterol levels [28,29,30,31,32,33]. In addition, LA has received more attention, and has been found to have a lipid-lowering effect, particularly in increasing HDL levels [34,35]. Thus, the present study aimed to investigate the potential of Fraction-7 of *X. muta* extract, which later proposed using LA as a lipid-lowering compound targeting *SR-BI* protein expression.

## 2. Results

### 2.1. Cytotoxicity of Diethyl Ether Fractions of X. muta

All seven fractions of *X. muta* were tested via the 3-(4,5-dimethylthiazol-2-yl)-5-(3-carboxymethoxyphenyl)-2-(4-sulfophenyl)-2H-tetrazolium (MTS) assay (Table 1). Fractions 1, 2, 5, 6, and 7 showed a nontoxic effect on HepG2 cells. At a concentration of 100 μg/mL of treatment, there was no significant effect of cell inhibition as more than 50% of the cells in the area were still viable. However, Fractions 3 and 4 showed a toxicity effect at lower concentrations, in which the fractions inhibited 50% of the cells.

### 2.2. SR-BI Transcriptional Activation by Diethyl Ether Fractions of X. muta

**Figure 1:**
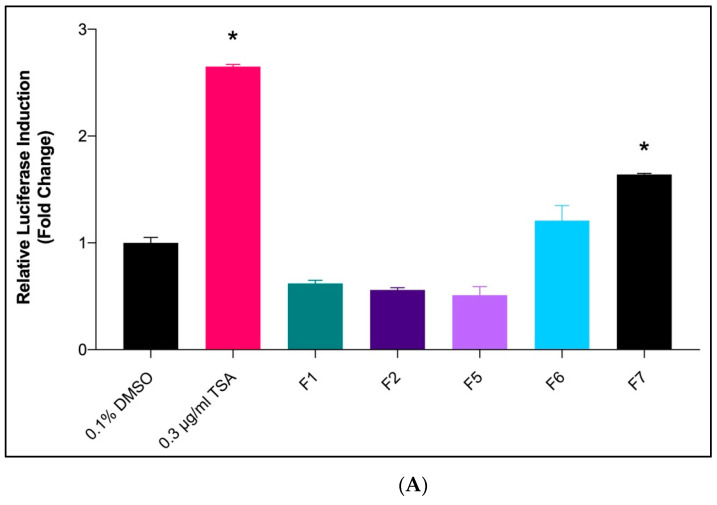

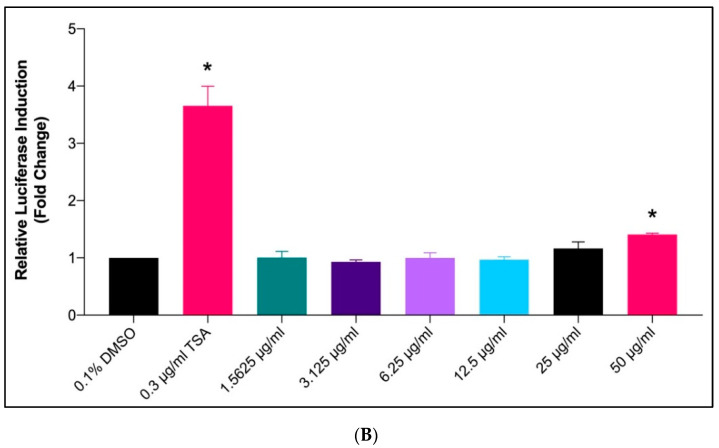
The effect of *X. muta* diethyl ether fractions (**A**) and Fraction-7 of *X. muta* (**B**) on *SR-BI* promoter activity in HepG2 cells. Results are expressed as mean ± SD (*n* = 3). * *p* < 0.05 vs. 0.1% DMSO. DMSO: dimethyl sulfoxide; TSA: Trichostatin A; F: fraction.

Based on the data on cytotoxicity, Fractions 1, 2, 5, 6, and 7 of *X. muta* were nontoxic to HepG2 cells. Hence, these fractions were selected to determine their potential activity as *SR-BI* promoters via a luciferase assay system. Based on the luciferase assay performed (Figure 1A), Fraction 7 and Fraction 6 of diethyl ether of *X. muta* were able to induce *SR-BI* gene expression at their effective concentration of 50 μg/mL. The highest fold change was recorded for Fraction-7 (*p* < 0.05) as compared to the negative control (0.1% DMSO), followed by Fraction 6.

Fraction-7 of *X. muta* was selected as it showed a significantly higher fold change than other fractions. Figure 1B shows that 50 μg/mL was the most potent concentration (*p* < 0.05) of Fraction-7 of *X. muta* on the *SR-BI* promoter activity. Hence, this concentration was selected for real-time polymerase chain reaction (Rt-PCR).

### 2.3. GC-MS of Fraction-7 of X. muta

**Figure 2:**
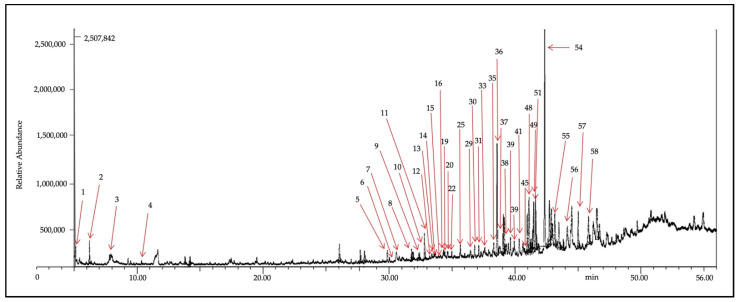
Sterol and fatty acids composition in Fraction-7 of *X. muta* detected by GC-MS with relative time.

The composition of Fraction-7 of *X. muta* further analyzed using gas chromatography-mass spectrometry (GC-MS) is shown in Figure 2, where 58 compounds were detected. Details of the sterol and fatty acid composition in Fraction-7 of *X. muta* were presented in Supplementary Table in the supplementary files.

### 2.4. SR-BI Transcriptional Activation by Potential Fatty Acids

**Figure 3:**
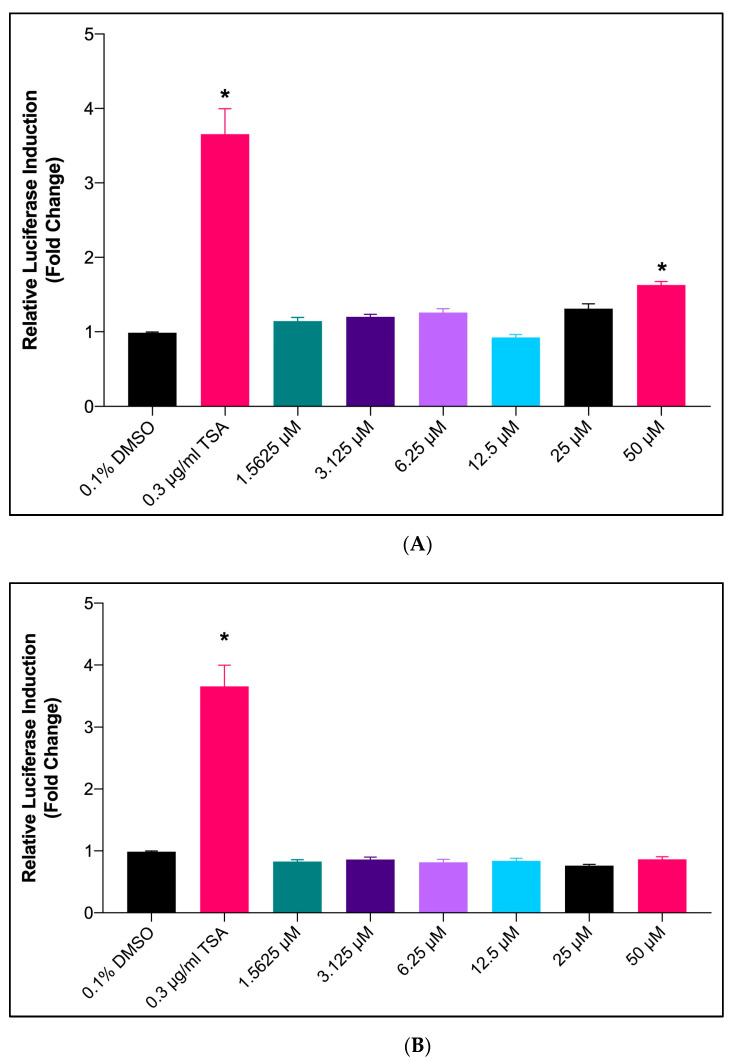
The effect of linoleic acid (**A**) and Hexadecenoic acid (**B**) on *SR-BI* promoter activity in HepG2 cells. The graph shows the relative fold induction of the transcriptional activity of *SR-BI* promoter with treatments using six different concentrations of compounds compared to negative control (0.1% DMSO) and positive control (TSA). Results are expressed as mean ± SD (*n* = 3). * *p* < 0.05 vs. 0.1% DMSO. DMSO: dimethyl sulfoxide; TSA: Trichostatin A.

As shown in Figure 3A, the treatment of LA produced a significant increase (*p* < 0.05) in the *SR-BI* promoter activity at a concentration of 50 μM compared to the negative control (0.1% DMSO). However, treatment with LA at concentrations from 1.56 μM to 25 μM did not produce any significant difference in *SR-BI* promoter activity compared to the negative control.

As shown in Figure 3B, the treatment using hexadecenoic acid did not produce any significant increase in the transcriptional activity of the *SR-BI* promoter at the concentration range tested as compared to the negative control.

### 2.5. Isolation and Quantification of Total RNA

Total cellular RNA was isolated from HepG2 cells after treatment. DNase treatment was carried out for all isolated RNA samples to ensure that the samples were free from DNA contamination. In the present study, the presence of two distinguished bands representing 28S and 18S rRNA indicated that the total RNA was highly intact. The quantification of RNA at the absorbance A_260_/A_280_ ratio of the isolated RNA was between 1.8 and 2.0, signifying that RNA samples were pure.

### 2.6. Real-Time PCR and SR-BI Gene Expression

#### 2.6.1. Optimization of Semi-Quantitative Rt-PCR Reaction

Optimization of semi-quantitative Rt-PCR on *SR-BI* and *β-actin* genes was carried out to determine the optimal annealing temperature for the primer sets used in the present study. The specific annealing temperature for both genes was determined by conducting PCR using temperatures ranging from 50 °C to 60 °C. Based on the melt curve produced, there was only a single peak reached, indicating that the primer set designed was very specific to the targeted genes. In this study, all the primer sets generated a single product with a specific melting temperature for both *SR-BI* and *β-actin* genes at 56 °C. Therefore, the optimal annealing temperature was fixed at 56 °C for both genes.

#### 2.6.2. Semi-Quantitative Rt-PCR Amplification Efficiency

Evaluation of semi-quantitative Rt-PCR amplification efficiency is very important in gene quantification procedures because it affects the accuracy of the calculated expression results and is critically influenced by the components of PCR. The efficiency of each primer set was determined by performing serial dilutions of the DNase-treated total RNA samples, starting from 500 ng of the RNA sample. In the present study, the efficiency of the *SR-BI* primer was 110%. The values obtained were within the recommended efficiencies of 90% to 110%, which signifies the implication efficiency of the primer sets on amplification. In addition, the correlation coefficients of *β-actin* and *SR-BI* were 0.978 and 0.963, respectively, which point out the linear relationship between the two variables tested. Thus, the amplification conditions used in this study were suitable for downstream studies.

#### 2.6.3. mRNA Expression of *SR-BI* Gene upon Treatment with Fraction-7 and LA

**Figure 4:**
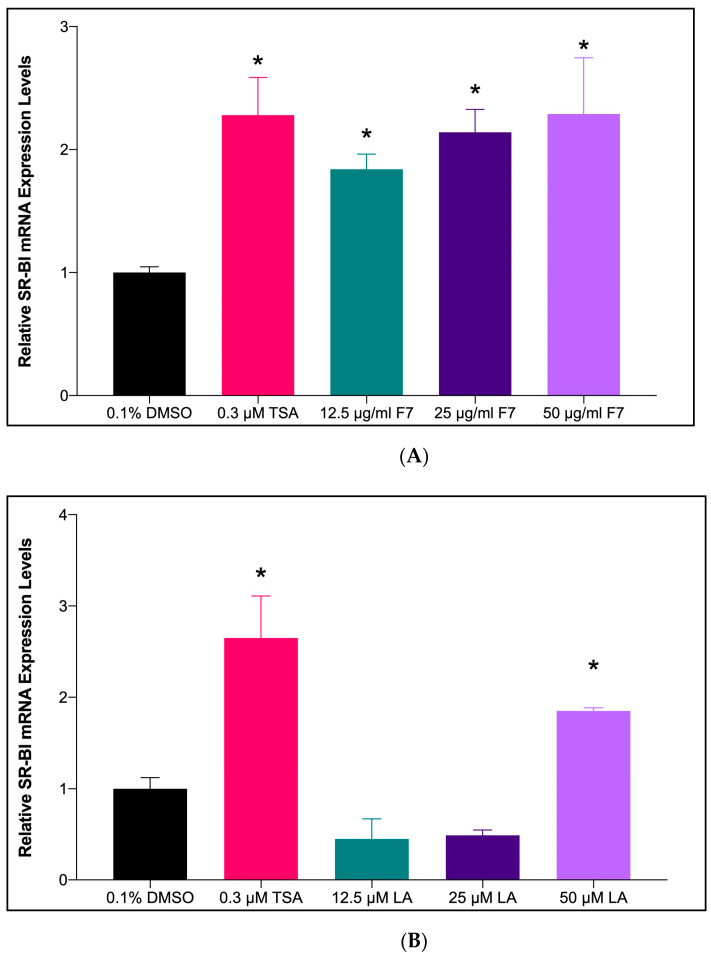
The effect of *X. muta* Fraction-7 (**A**) and LA (**B**) on *SR-BI* mRNA expression of HepG2 cells. 0.1% DMSO (negative control), 0.3 μM TSA (positive control), Fraction-7 of *X. muta* (12.5, 25, and 50 μg/mL), and LA (12.5, 25, and 50 μM). Graphical representation of the data normalized to *β-actin*. Data are presented as mean ± SD (*n* = 3). * *p* < 0.05 vs. 0.1% DMSO. DMSO: dimethyl sulfoxide; TSA: Trichostatin A; F: fraction; LA: Linoleic acid.

In the present study, treatment with three different concentrations of Fraction-7 of *X. muta* (12.5, 25, and 50 μg/mL) showed significantly increased mRNA expression of the *SR-BI* gene (*p* < 0.05) (Figure 4A). Fraction-7 at a concentration of 50 μg/mL increased *SR-BI* transcription 2.29-fold compared to the negative control (F7: 2.29 ± 0.46 vs. 0.1% DMSO: 1.00 ± 0.07). No significant difference (*p* > 0.05) was seen in each concentration of *X. muta* Fraction-7 (12.5, 25, and 50 μg/mL) when compared to the trichostatin A (TSA) treatment, suggesting that the effect of three concentrations of Fraction-7 of *X. muta* in increasing mRNA expression of *SR-BI* was comparable with the effect of TSA treatment.

In the present study, treatment with the highest concentration of LA (50 μM) showed significantly increased mRNA expression of the *SR-BI* gene (*p* < 0.05) (Figure 4B). LA at a concentration of 50 μM increased *SR-BI* transcription 1.85-fold compared to the negative control (LA: 1.85 ± 0.04 vs. 0.1% DMSO: 1.00 ± 0.16). The effect of the highest concentration of LA (50 μM) was comparable with the effect of TSA treatment.

#### 2.6.4. Time Lapse for *SR-BI* Expression of HepG2 Cells Treated with Fraction-7 and LA

**Figure 5:**
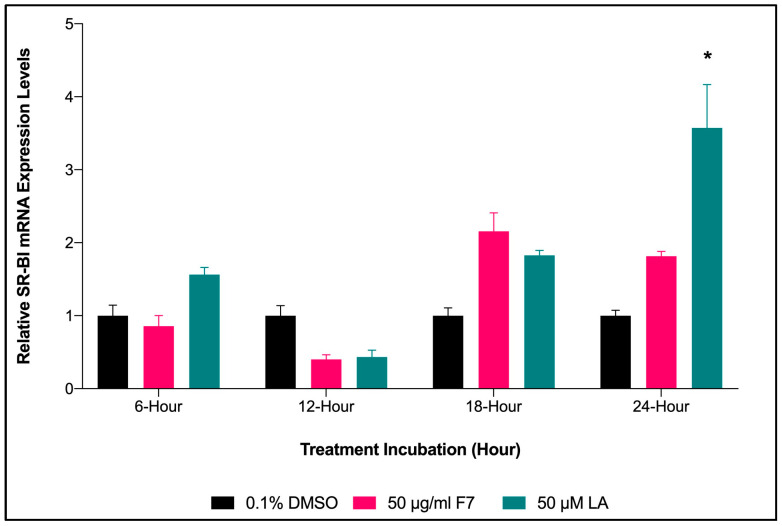
The effect of time for Fraction-7 of *X. muta* and LA on *SR-BI* mRNA expression on HepG2 cells. Graphical representation of the data shows the relative *SR-BI* expression on HepG2 cells treated with 0.1% DMSO, Fraction-7 of *X. muta* (50 μg/mL), and LA (50 μM). Graphical representation of the data normalized to *β-actin*. Data are presented as mean ± SD (*n* = 3). * *p* < 0.05 vs. 0.1% DMSO. DMSO: dimethyl sulfoxide; TSA: Trichostatin A; F: fraction; LA: Linoleic acid.

In the present study, Fraction-7 of *X. muta* showed a trend of an increase in *SR-BI* expression with an increase in the incubation time of the treatment. For Fraction-7, the expression was highest at 18 h of incubation, while 24 h of incubation showed a significant increase in *SR-BI* expression for LA (*p* < 0.05) compared to 0.1% DMSO (negative control) (Figure 5). However, no significant difference was seen among the study groups in incubation treatments at 6, 12, and 18 h.

### 2.7. HDL-C Uptake and Protein Cellular Localization

#### 2.7.1. HDL-C Uptake by HepG2 Cells Treated with LA

**Figure 6:**
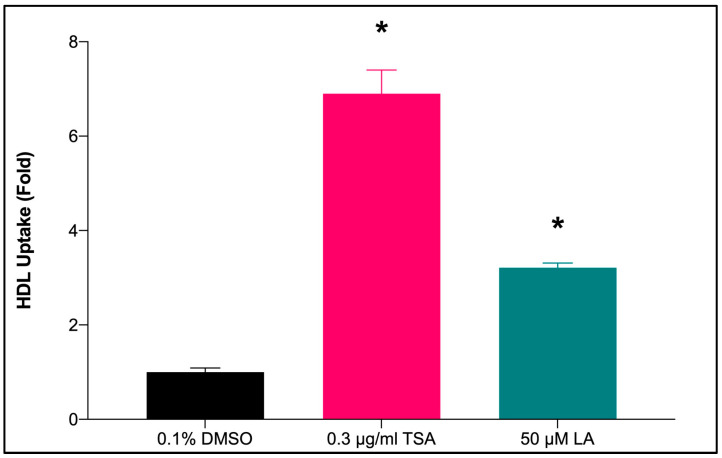
The effect of LA treatment on treated HepG2 cells on HDL uptake. Graphical representation of the data shows the relative Dil-HDL fluorescent intensity of HepG2 cells treated with 0.1% DMSO, 0.3 μM TSA, and 50 μM LA. Data are presented as mean ± SD (*n* = 3). * *p* < 0.05 vs. 0.1% DMSO. DMSO: dimethyl sulfoxide; TSA: Trichostatin A; LA: Linoleic acid; HDL: High-density lipoprotein.

Figure 6 shows that the treatment with LA at 50 μM significantly enhanced (*p* < 0.05) the uptake of HDL into the HepG2 cells by 3.21-fold (LA: 3.17 ± 0.04 vs. 0.1% DMSO: 0.99 ± 0.02) as compared to 0.1% DMSO (negative control). The results closely corresponded to the levels of mRNA and protein content of *SR-BI* in HepG2 cells treated with the same compound.

#### 2.7.2. *SR-BI* Protein Levels in Treated HepG2 Cell Lines

**Figure 7:**
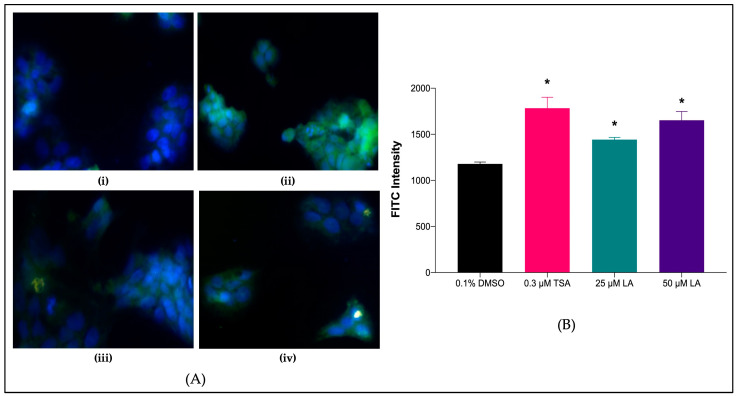
Immunocytochemistry staining for the effect of 25 μM and 50 μM LA on HepG2 cell lines (**A**). Graphical representation of FITC intensity of *SR-BI* receptor protein on HepG2 cell lines (**B**). Representative fluorescent microscopic images showed that *SR-BI* receptor protein expression (green) and nucleus (blue) in immune-stained HepG2 cells. (i) 0.1% DMSO (negative control); (ii) 0.3 μM TSA (positive control); (iii) 25 μM LA; (iv) 50 μM LA; 20× magnification. Data are presented as mean ± SD (*n* = 3). * *p* < 0.05 vs. 0.1% DMSO. DMSO: dimethyl sulfoxide; TSA: Trichostatin A; LA: Linoleic acid; FITC: Fluorescein isothiocyanate.

The effects of LA on the expression of *SR-BI* were further validated by determining the levels of the protein content and localization of *SR-BI* present in the treated HepG2 cells using immunocytochemistry analysis (Figure 7). The nuclear and chromosome counterstain 4′,6-diamidino-2-phenylindole (DAPI) emits blue fluorescence upon binding to the nucleus components of the fixed HepG2 cells. Through visual observation of the immunofluorescent intensity, those treated with LA showed considerably higher green fluorescence intensity than the negative control (0.1% DMSO) (Figure 7Aiii,iv). These results showed a significant increase in *SR-BI* protein expression after treatment with LA compared to the negative control (*p* < 0.05) (Figure 7B).

## 3. Discussion

Limited studies have investigated the impact of marine sponges and their bioactive compounds on lipid-lowering activity and cardiovascular parameters in in vitro hypercholesterolemic models. A few studies have indicated that bioactive compounds isolated from marine sponges show pharmacological effects that include anti-microbial, anti-inflammatory, and anti-tumor properties [14,21,22,26]. To the best of our knowledge, this current study is the first to show that treatments with *X. muta* fraction and LA (an active compound from *X. muta*) increased *SR-BI* transcriptional activity and mRNA expression of the *SR-BI* gene and enhanced HDL uptake in HepG2 cells. This study also demonstrated that treatment with Fraction-7 of *X. muta* and LA showed no cytotoxicity effect in the MTS assay. Regarding the phenolic compounds of *X. muta*, a total of 58 compounds were identified from the Fraction-7 of *X. muta,* including 9,12-octadecadienoic acid (LA), which was used in the current study.

In the present study, the cytotoxicity of the fractions of *X. muta* was examined first before determining their ability to increase *SR-BI* promoter activity and mRNA expression of the *SR-BI* gene. The cytotoxicity of the fractions from *X. muta* obtained by successive solvent extraction indicated by the IC_50_ against HepG2 cells was higher than 50 μg/mL (Table 1). From the data (Table 1), Fractions 3 and 4 were considered cytotoxic (<50 μg/mL). Fractions 1, 2, 5, 6, and 7 produced no cytotoxic effect (>50 μg/mL) and were selected for further investigation in the *SR-BI* promoter expression assay. This finding is in line with the previous studies by Azemi et al. [36] and Dung et al. [37].

*SR-BI* mediates the selective uptake of cholesterol esters from HDL [13,38]. Upregulation of *SR-BI* gene expression in the liver may produce anti-atherosclerosis by increasing the catabolism of the lipid from HDL and atherogenic lipoprotein [13]. It has been shown that HDL plays a significant role in RCT by removing plasma cholesteryl ester and accumulated cholesterol ester along the blood circulation to the liver, thus reducing the risk of atherosclerosis development. However, *SR-BI* not only mediates HDL cholesteryl ester uptake but also stimulates free cholesterol efflux from cells into HDL, suggesting that *SR-BI* in the blood vessel wall might play a pivotal role in cholesterol removal [13,39]. In addition, studies have demonstrated that overexpression of hepatic *SR-BI* is associated with the reduction of plasma HDL cholesterol levels transported into the bile [13,40,41]. In the current study, Fractions 1, 2, 5, 6, and 7 of *X. muta* proceeded to *SR-BI* transcriptional activity via luciferase assay. From the present data, it was shown that Fraction-7 of *X. muta* exhibited a significantly higher luciferase fold change compared to the negative control (0.1% DMSO). The effect of Fraction-7 on the increasing luciferase fold change was comparable with the effect of TSA (positive control). Fraction-7 was tested further using a luciferase assay at six different concentrations (1.5625–50 μg/mL). From the present data, Fraction-7 of *X. muta* at a concentration of 50 μg/mL showed the highest (*p* < 0.05) luciferase fold change compared to the negative control.

In the present study, GC-MS analysis of the Fraction-7 (most effective fraction) of *X. muta* showed the presence of 58 bioactive compounds, which include essential polyunsaturated fatty acids, particularly LA. Previous studies have demonstrated that LA reduces plasma cholesterol and plays a pivotal role in preventing cardiovascular diseases [30,42]. Based on GC-MS analysis and luciferase assay, LA was selected for further use in the present study due to the highest activity in luciferase assay as compared to unsaturated fatty acid hexadecenoic acid. In the present study, both Fraction-7 of *X. muta* and LA were treated in HepG2 cells to determine their effects on the mRNA expression of the *SR-BI* gene. From the data in the present study, the highest doses of Fraction-7 of *X. muta* (50 μg/mL) and LA (50 μM) showed a significant increase in mRNA expression of the *SR-BI* gene compared to the negative control. The effect of both treated groups was comparable with the effect of the positive control (0.3 μM TSA). The present finding of LA was in line with our previous preliminary in vitro study on the effect of LA on *SR-BI* expression [43].

Due to limited samples of Fraction-7 of *X. muta* in the present study, only LA was used for the HDL uptake assay and immunocytochemistry. LA was suggested for this purpose as this compound was found to be present in the Fraction-7 of *X. muta* via previous GC-MS analysis data. In the HDL uptake assay, treatment with LA and TSA at 50 μM and 0.3 μM significantly increased the uptake of HDL into the HepG2 cells by 3.21-fold and 6.90-fold, respectively, as compared to 0.1% DMSO (negative control). No significant difference was seen between both treated groups, showing the comparable effect of both treatments in increasing HDL uptake. Previous studies have reported that treatment with LA enhanced HDL levels in human [29,30] and animal [44] studies. In this study, increased HDL uptake in the LA-treated HepG2 cells was attributed to increased mRNA expression of the *SR-BI* gene. In addition, an increase in HDL uptake can be related to the absence of CC-chemokine ligand 2 (CCL2). According to Sun et al. [45], the presence of CCL2 can suppress the internalization of HDL, making it unable to further transfer cholesterol to the liver [45]. This was detected by [^3^H]-labeled cholesterol, where cholesterol efflux significantly decreased in the time-course experiment within 24 h [45]. Shen et al. showed an increase in cholesterol efflux after labeling the cholesterol with [^3^H] [46]. In a previous study [46], the level of cholesterol efflux was measured after treatment with a soluble epoxide hydrolase inhibitor, which can lead to an increase in ABCA1 expression. An increase in ABCA1 can increase cholesterol efflux.

An immunocytochemistry study was conducted to determine the effect of the treatments in upregulating *SR-BI* availability after translation. Treatments successfully showed increased *SR-BI* mRNA expression by referring to the green luminescence on the cell surface. The upregulation of *SR-BI* expression was determined by using a specific antibody so that the presence of *SR-BI* after treatment could be viewed qualitatively. From the results, the availability of *SR-BI* can be seen within the cytoplasm and on the surface of the cells, based on green luminescence. In the present study, both concentrations (25 μM and 50 μM) of LA-treated HepG2 cell lines showed higher green fluorescence intensity compared to the negative control (0.1% DMSO). Therefore, it was confirmed that *SR-BI* expression was upregulated after treatment with LA in HepG2 cells.

## 4. Materials and Methods

### 4.1. Sample Collection

**Figure 8:**
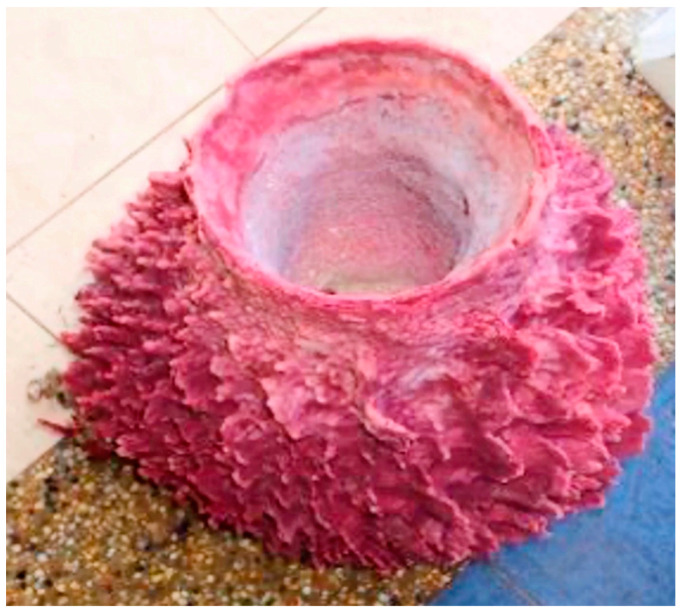
*Xestospongia muta* (Schmidt, 1870) sampled by Institute of Marine Biotechnology, Universiti Malaysia Terengganu, Malaysia.

Samples of *X. muta* (Figure 8) were collected via scuba diving at a depth of 5–10 m at Telok Belanga from the Archipelago of Bidong Island Terengganu, Malaysia, which is located at N 05°36.656′ E 103°04.024′. The samples were photographed in situ for better species characterization. The sponge species were authenticated by Dr. Jasnizat Saidin from the Institute of Marine Biotechnology (IMB), Universiti Malaysia Terengganu (UMT), Malaysia. A voucher specimen, SPO1015010, was deposited at the Biota Depository, IMB, UMT.

### 4.2. Preparation of the X. muta Extract

The sponge extraction was carried out according to the methods previously described by Andriani et al. [13] and Azemi et al. [36]. Freshly collected sponged specimens were cleaned from debris, chopped, and stored at −80 °C before lyophilization using a freeze dryer (FD-550, EYELA, Japan) to remove water. To obtain a methanolic crude extract of *X. muta*, 1.25 kg of the *X. muta* powder was exhaustively macerated with absolute methanol in the ratio of 10 g of dried sample to 100 mL of methanol. This procedure was repeated three times to maximize the extraction. After maceration, the solution was filtered and evaporated using a vacuum rotary evaporator at 40 °C under reduced pressure to obtain methanolic crude extract. This extraction yielded approximately 51.795 g of dried crude methanol extract of *X. muta* (the yield was 4.1% (*w*/*w*)). Dry methanol crude extract was reconstituted in diethyl ether, transferred into a separation funnel, and combined with distilled water in a 2:1 ratio of diethyl ether to water, respectively [36,47]. The separation funnel was then shaken up vigorously before the active compounds were allowed to settle down according to their polarity. The partitioning process was repeated at least three times or until the diethyl ether phase turned colorless. To further obtain the polar compounds from the separation, *n*-butanol was added to the separation funnel in exchange for diethyl ether and shaken vigorously together with the distilled water used in the nonpolar extraction beforehand. Both diethyl ether and *n*-butanol fractions were dried completely using a vacuum rotary evaporator, and the metabolic profile was checked via thin-layer chromatography (TLC) for the isolation of bioactive compounds from the sample.

### 4.3. Preparation of X. muta Fractions

Column chromatography was further used to separate the molecules in the fractions so that isolation and characterization could be performed efficiently. The method described by Andriani et al. [13] was adapted to carry out column chromatography. The diethyl ether fraction of the sample was subjected to a series of chromatographic columns (23 cm × 3 cm) using hexane and ethyl acetate and subsequently followed by ethyl acetate and methanol in different ratios as solvent systems, while silica gel 60 (0.040–0.063 mm; Merck, Germany) was used as the stationary phase. Fractions of about 100 mL each were collected, evaporated, labeled, and monitored by TLC using hexane:ethyl acetate (7:3) as a solvent system. The fractions showing the same TLC pattern were combined, resulting in 7 fractions, namely Fractions 1–7.

### 4.4. Cell Culture

HepG2 cell lines were obtained from the American Type Culture Collection (Rockville, MD, USA) and maintained in a culture flask, as described previously [13]. The HepG2 cells were kept in minimum essential media (MEM) (Sigma, St. Louis, MO, USA) with 10% fetal bovine serum (ICN Biomedicals, Aurora, OH, USA) and 1% penicillin–streptomycin at 37 °C under 5% CO_2_.

### 4.5. Cytotoxicity Study Fraction by MTS Assay

The MTS assay was used to determine cell viability. HepG2 cells with 80–90% confluency were seeded in a 96-well plate at 8 × 10^3^ cells/wells, 24 h before treatment. The samples were dissolved in 1 mL of 0.1% DMSO and were subsequently diluted twofold ranging from 1.56 μg/mL to 100 μg/mL. Cells treated with 0.1% DMSO act as the negative control, whereas cisplatin (Sigma Aldrich, St. Louis, MO, USA) acts as the positive control. The 96-well plate was incubated for 72 h at 37 °C in the presence of 5% CO_2_. After incubation, 20 μL of MTS solution was added to each well under dark conditions, and the plates were incubated for 90 min. The absorbance at 495 nm was read on a microplate reader in which the intensity of light produced was directionally proportional to the number of cells viable in the well plate.

### 4.6. SR-BI Transcriptional Activity of X. muta Fractions via Luciferase Assay

The induction of *SR-BI* transcriptional activity was conducted via luciferase assay according to the method described in previous studies [8,48]. Prior to the day of treatment, 3 × 10^4^ cells/well of the stable HepG2 cell line containing *SR-BI* promoters were seeded in a 96-well plate at a volume of 100 μL of complete medium without pen-strep in each well. The cells were incubated for 24 h at 37 °C in a CO_2_ incubator until the confluency reached 60–80%. After 24 h, the media was discarded and replaced with complete media containing 1% pen-strep and 50 μg/mL of each *X. muta* fraction (Fractions 1–7). The cells were also treated with 0.1% DMSO (negative control) and 0.1 μg/mL TSA (positive control). TSA is a natural derivative of dienohydroxamic acid derived from the fungal metabolite, which exerts antidiabetic [49,50], anti-inflammatory [51,52], lipid-lowering [53], and antioxidant [54,55] properties. It is known as an inhibitor of histone deacetylases and one of the most effective agents with validated targets that prevent the progression of hypercholesterolemia [53,56,57].

After 24 h of treatment, 20 μL of Cell Titer-Flour was added to all wells, which were then covered with aluminum foil and incubated for 30 min. The absorbance was read at 492 nm. Then, 90 μL of ONE-Glo reagent was added and mixed well to ensure that the reagent and the sample were homogenized evenly. After 3 min of incubation, luciferase activity was determined using a Glomax luminometer (Promega). The cytotoxicity results using the MTS assay were used to normalize the luciferase results to obtain the actual results of the luciferase activity of the treatment. The luciferase activity was expressed as the number of fold changes.

### 4.7. SR-BI Transcriptional Activity of Fraction-7 of X. muta via Luciferase Assay

Fraction-7 was chosen (highest luciferase activity fold-change) to further determine its potential activity as *SR-BI* promoters via luciferase assay against HepG2 cells transfected with the *SR-BI* gene. Two-fold concentrations ranging from 1.56 μg/mL to 50 μg/mL were used to determine the highest concentration inducer to *SR-BI* transcriptional activity.

### 4.8. Gas Chromatography-Mass Spectrometry (GC-MS) Analysis of Fraction-7 of X. muta

GC-MS analysis was carried out to identify and quantify the sterol composition of Fraction-7. The identification was carried out according to the previous study by Azemi et al. [36] using the Shimadzu QP2010SE Ultra equipped with Wiley Library software. Separation of analytes by gas chromatography was carried out using a BP5MS GC column (30 m × 0.25 mm, 0.25-μm film thickness). The oven was programmed as follows: initial temperature was 50 °C (hold 1 min) to 300 °C (rate 5 °C/min, hold 5 min), the carrier gas was helium gas (99.99% purity), and the flow rate was 4.9 mL/min. Significant mass spectrum operating parameters were ionization voltage, 70 eV; ion source temperature, 200 °C; and scan mass range, 50–600 u. The relative percentage amount of each component was calculated by comparing its average peak area to the total areas. Interpretation of GC-MS was conducted using a database of the National Institute Standard and Technology (NIST) having more than 62,000 patterns.

### 4.9. Induction of SR-BI Transcriptional Activity by Potential Fatty Acids

Based on the GC-MS results of Fraction-7 of *X. muta*, the most potent compounds related to cardiovascular diseases were 9,12-octadecadienoic acid (LA) and hexadecenoic acid (palmitoleic acid). These selected compounds were chosen based on previous studies that showed effectiveness-related bioactivity [34,35,58,59]. However, due to an insufficient amount of Fraction-7 of *X. muta* obtained, LA and hexadecenoic acid were used in the current study, which were commercially available compounds supplied by Sigma Aldrich (St. Louis, MO, USA). Hence, the selected compounds were tested with luciferase assay to select the most active compound in increasing the transcription of *SR-BI*. The assay was carried out in six different concentrations, ranging from 1.56 μM to 50 μM. The induction of *SR-BI* transcriptional activity by these two compounds was performed according to the protocols in Section 4.6.

### 4.10. Treatment of HepG2 Cells with Fraction-7 of X. muta

#### 4.10.1. Transfection of HepG2 Cells with *SR-BI* Promoter

HepG2 cells were transfected with the *SR-BI* gene. The *SR-BI* gene was ligated into pGL4.17 [luc/Neo] plasmid at NheI and HindIII, resulting in the activation of the *luc2* gene in the plasmid. To confirm that the *SR-BI* gene was already transfected into HepG2 cells, extraction of total DNA from transfected HepG2 cells was done using a DNAeasy Blood & Tissue kit (Cat. No./ID: 69504; Qiagen, Hilden, Germany) for cell and tissue following the manufacturer’s protocols. HepG2 cells in the flask were detached using 500 μL of trypsin. Then, the sample was removed into a sterile tube and centrifuged at 3000× *g* for 5 min. The supernatant was discarded, and the cell pellet was resuspended in 200 μL of phosphate buffer saline. A total of 20 μL of proteinase K and 200 μL of buffer AL were added to the tube, and the tube was vortexed for 10 s and incubated for 10 min at 56 °C. After that, 200 μL of ethanol was added to the tube, and it was vortexed for 15 s. The mixture was then transferred into a QIAamp mini spin column, and it was centrifuged at 6000× *g* for 1 min. The supernatant was discarded, and 500 μL of buffer AW1 was added to the spin column. The spin column was centrifuged at 600× *g* for 1 min, and the supernatant was discarded. Then, 500 μL of buffer AW1 was added to the spin column and centrifuged at 20,000× *g* for 3 min. The supernatant was discarded, and 100 μL of buffer AE was added to the spin column and incubated at room temperature for 1 min. The spin column was later centrifuged, and the supernatant containing DNA was collected. The DNA from HepG2 cells was subjected to PCR to amplify the *SR-BI* and *luciferase* genes, and 5 μL of 10× PCR buffer was added into a sterile tube, followed by 1.5 μL of 25 mM of MgCl_2_, 0.5 μL of dNTP mix, 1 μL of forward primer, 1 μL of reverse primer, 1 μL of genomic DNA, and 0.2 μL of 5 U/μL of Taq polymerase. A total of 14.8 μL of distilled water was added to the tube to make a final volume of 25 μL. The sequences of the forward and reverse primers used are listed in Table 2. The thermocycler was set for the PCR as follows: initial denaturation step at 94 °C for 1 min and 40 cycles of denaturation at 94 °C for 30 s, primer annealing at 62 °C for 30 s, elongation at 72 °C for 30 s, and final elongation step at 72 °C for 5 min. The PCR product was then subjected to gel electrophoresis on 1% agarose gel stained with ethidium bromide. The electrophoresis was conducted at 90 V for 40 min.

#### 4.10.2. Treatment of transfected HepG2 Cells with Fraction-7 of *X. muta* and LA

A total of three concentrations of Fraction-7 and LA were used. HepG2 cells cultured in a 25-cm^3^ flask were treated with a concentration of 12.5 μg/mL, 25 μg/mL, and 50 μg/mL for Fraction-7 of *X. muta* and 12.5 μM, 25 μM, and 50 μM for LA in 24 h.

### 4.11. Extraction of Total RNA

#### 4.11.1. RNA Extraction from HepG2 Cells

The RNA of the treated HepG2 cells was isolated using 1 mL of TRIzol^®^ (Cat. No.: TR118; Molecular Research Center, Inc., Cincinnati, OH, USA) reagent. Briefly, the cells were homogenized and incubated for 5 min at room temperature for complete dissociation of the nucleoprotein complex. The homogenate was transferred into a sterile 1.5 mL microcentrifuge tube. After that, 100 μL of 1-bromo-3-chloropropane (BCP) was added to the tube and vortexed vigorously for 15 s. Then, the mixture was placed at 4 °C for 15 min and centrifuged at 12,000× *g* for 15 min at 4 °C. Three layers consisting of the clear upper aqueous phase, an intermediate phase, and a lower red phenol-protein phase were formed after centrifugation. The upper clear aqueous phase was carefully transferred to a clean 1.5 mL microcentrifuge tube and precipitated by adding 500 μL of isopropanol. The tube was then incubated at room temperature for 10 min, followed by centrifugation at 12,000× *g* for 8 min at 4 °C. The supernatant was gently decanted, and the RNA pellet was washed with 80% (*v*/*v*) ethanol by centrifuging at 12,000× *g* for 5 min at 4 °C. The ethanol was decanted carefully, and the washing step was repeated twice. After the washing step, the RNA pellet was air-dried for 5 min and dissolved in sterile distilled water. Finally, the RNA sample was stored at −80 °C for downstream application.

#### 4.11.2. RNA Extraction from Tissue

The procedure for total RNA extraction using *TransZol* Up (Cat. No.: ER501-01; Beijing, China) was applied to the liver tissue, which is very rich in RNases. One gram of the healthy rat’s liver tissue was grounded in liquid nitrogen until it became powder. About 50–100 mg of powdery tissue was transferred to a 1.5 mL microcentrifuge tube, and 1 mL of *TransZol* Up was added to homogenize the tissue by pipetting up and down. The tube was then incubated for 5 min. Then, 0.2 mL of chloroform was added to the tube, shaken vigorously, and incubated for another 3 min. Then, the mixture was centrifuged at 10,000× *g* for 15 min at 4 °C. Three layers consisting of a clear upper aqueous phase that contained the RNA, an intermediate phase, and a lower red phenol-protein phase were formed after centrifugation. The volume of the aqueous upper phase consists of 50–60% of the *TransZol* Up reagent. The upper clear aqueous phase was carefully transferred into a clean 1.5 mL microcentrifuge tube and precipitated by adding 100% ethanol. The mixture was then transferred to a spin column and centrifuged at 12,000× *g* for 30 s at room temperature. The filtrate was discarded, and 500 μL of buffer WB9 (wash buffer) was added and centrifuged at 12,000× *g* for 2 min at room temperature to completely remove the remaining ethanol and then air-dried. Finally, the spin column was placed in a clean 1.5 mL microcentrifuge tube, and 100 μL of RNase-free water was added. The tube was incubated at room temperature for 1 min and centrifuged at 12,000× *g* for 1 min to elute the RNA. For stable and longtime storage, the extracted genomic RNA was stored at −80 °C.

#### 4.11.3. Quantification of Total RNA

The concentration and purity of the isolated RNA were determined by measuring the absorbance at 260 nm and 280 nm using a microvolume measurement platform (Bio-Drop, Biochrom Ltd., Cambridge, UK). Only high-quality samples with an A_260_/A_280_ ratio of 1.8–2.0 were used for further experiments. The integrity of the isolated RNA was determined by electrophoresis on 1% (*w*/*v*) agarose gel electrophoresis. First, the agarose gel was prepared by melting 0.36 g of agarose powder in 30 mL of 1× Tris–Borate–EDTA (TBE). Then, 1 μL of ethidium bromide was added to the gel and left to cool. The samples were mixed with RNA loading buffer (6×) and subjected to gel electrophoresis at 90 V for 40 min. The gel was visualized using GeneSnap software on the Gene Genius Bio Imaging System (Syngene UK, Cambridge, UK).

#### 4.11.4. DNase Treatment of Total RNA

The total RNA extracted was pretreated with RQ1 RNase-free DNase (Cat. No.: M6101; Promega, WI, USA) to eliminate traces of DNA contamination. The reaction was performed in a 10 μL mixture containing 500 μg of total RNA, 1 μL of RQ1 RNase-free DNase buffer 5×, and 2 μL of RQ1 RNase-free DNase. The mixture was incubated at 37 °C for 60 min, followed by 65 °C for 10 min with the addition of 1 μL of RQ1 DNase stop solution to terminate the enzyme activity.

### 4.12. Rt-PCR of SR-BI Gene Expression

The levels of mRNA expression of the *SR-BI* gene were measured by Rt-PCR analysis using the iScript^TM^ One-Step RT-PCR kit with SYBR^®^ Green (Cat. No.: #1708892; Bio-Rad Laboratories, Hercules, CA, USA) according to the manufacturer’s protocols. Primers for the *SR-BI* gene were designed using Primer3 software. *β-actin* gene was used as an internal control. A total of 50 ng of DNase-treated RNA was reverse transcribed into first-stranded cDNA at 50 °C for 20 min. This was followed by PCR amplifications using an initial temperature of 95 °C for 5 min and 40 cycles of 95 °C for 30 s, 59 °C for 30 s, and 72 °C for 30 s. This was followed by 95 °C for 1 min, 55 °C for 1 min, and 55 °C for 0.05 s. The average threshold cycle (Ct) for each treatment group was calculated from the experimental analysis in triplicate. The mRNA expression levels of *SR-BI* were determined using Ct values normalized to the internal control. An internal control of the *β-actin* gene was used in a separate tube to normalize the expression. The primers used are shown in Table 3.

The relative standard curve method was used to quantify gene expression. Equal aliquots of RNA from each sample were pooled together. This RNA was serially diluted twofold with sterile water to produce a seven-point standard curve. The slope of the standard curve indicates the efficiency of the Rt-PCR reaction. For these experiments, assays were considered acceptable if there was no more than a cycle threshold (Ct) difference of 0.5 between the values of a triplicate and the standard curve slope within the range (−3.2 to −3.6). A slope of −3.413 and an R2 value of 0.978 indicate 100% efficiency of the amplification reaction. A standard curve was included for each plate run, and each gene was studied. The mRNA expression level of specific genes was normalized to the geometric mean of the mRNA expression level of the reference gene, *β-actin*, which was compared analytically and used where appropriate. In the time-lapse study, the concentration of Fraction-7 and LA, which increased the expression of *SR-BI*, was used in time-course treatment. The concentration of 50 μg/mL was selected from the Fraction-7 of *X. muta,* and 50 μM was selected from LA as the potential concentration that has a significant effect on increasing the fold change. HepG2 cells cultured in a 25-cm^3^ flask were treated with a concentration of 50 μg of Fraction-7 of *X. muta* and 50 μM LA for 6, 12, 18, and 24 h.

### 4.13. Determination of HDL-C Uptake and SR-BI Protein Cellular Localization

HDL uptake was done to determine the effects of LA in regulating lipid uptake by HepG2 cell lines using the Dil-HDL uptake assay. *SR-BI* protein cellular localization was done to determine the levels of *SR-BI* mRNA expression on the translocation of *SR-BI* protein from the intracellular compartment to the surface of the cells. Results from current studies show that LA as a single compound shows a comparable result to Fraction-7 of *X. muta,* which contains more compounds, as stated in the GC-MS profile. Hence, the next step focused only on LA as a single compound to reduce complexity and identify a single active constituent. The positive result from Fraction-7 of *X. muta* may be contributed by the synergetic effect of other compounds, as the fraction contains more than one compound. Therefore, focusing on only one constituent will produce more reliable results.

#### 4.13.1. Determination of Lipid Uptake by Fluorometric Assay

HDL uptake activities were measured using commercially available HDL Uptake Assay Kits, Fluorometric (Cat. No.: ab204717, Abcam, Cambridge, UK) according to the manufacturer’s instructions. Briefly, 3 × 10^4^ cells were seeded in a 96-well plate with a transparent bottom and incubated overnight at 37 °C. After that, the cells were treated with 50 μM LA. After 24 h, the treatment in each well was discarded, and the cells were washed three times with 100 μL of assay buffer. The standard curve preparation included 1:100 dilution of the fluorescent-labeled HDL and was subsequently diluted twofold. Then, 100 μL of the prepared standard solution was added to empty wells in the microplate. The reaction mix was prepared by mixing fluorescent-labeled HDL with serum-free media in 1:49 dilution. The reaction mixture was then sterilized using a 0.22 μm syringe filter, and 100 μL of reaction mix was added to each sample, background control, and specificity control well and further incubated for 24 h. A well with 100 μL of serum-free media acted as a background control sample, whereas 10 μL of unlabeled HDL was used to measure the specificity of HDL uptake. After incubation, the medium was removed, and the cells were washed 4 times with 100 μL of assay buffer. The HDL uptake was measured using a Glomax luminometer (Promega) at Ex/Em = 540/575 nm based on the manufacturer’s instructions.

#### 4.13.2. Determination of *SR-BI* Protein Levels by Immunofluorescent Assay

Immunocytochemistry was performed to determine the presence of *SR-BI* protein on the surface of the HepG2 cells after being treated with LA. Two concentrations of LA were chosen to confirm the ability to increase *SR-BI* on the cell surface: 25 μM and 50 μM. HepG2 cells were seeded into a six-well plate with a glass slip of 2000 cells/100 μL. After 24 h, the cells were treated with one concentration of LA, which increased the mRNA expression by 24 h. After 24 h, the treatment was discarded and 1 mL of 4% (*w*/*v*) paraformaldehyde was added to each well, and the plate was incubated at room temperature for 25 min in the dark. After 25 min, 4% paraformaldehyde was removed, and the cells were washed three times with 1 mL of PBS, with 5 min for each wash. Then, 500 μL of Universal Protein Blocking Reagent (UPBR, Genetex, Alton Pkwy Irvine, CA, USA) was added to each well, and the plate was wrapped with aluminum foil and incubated at 27 °C for each hour. After the incubation, UPBR was discarded, 1 mL of 1× immunostain wash buffer (ISWB; Genetex, USA) was added, and the plate was agitated for 10 min. ISWB was discarded, and 500 μL of primary antibody (Scarb1 polyclonal antibody) was added to each well and incubated for 24 h at 4 °C. The antibody was diluted 500 times from the stock by a universal antibody dilution buffer (UADB; Genetex, USA). After 24 h of incubation, the antibody was discarded, and the cells were washed three times with ISWB for 10 min each. A secondary antibody (goat anti-rabbit IgG; PAB10822; Abnova, Taipei, Taiwan) diluted 1000 times was added, and the plate was incubated for 1 h at 4 °C. After 1 h, the antibody was discarded, and the cells were washed four times with 1 mL of ISWB for 10 min each. The plate was air-dried in the dark, and the cover slip was removed from the well. The cells were counterstained with DAPI to cover the entire coverslip. When the counterstained DAPI was dried, the coverslip was placed on a glass slide with the surface of the cells facing the glass slide mounted with a mounting medium. The stained cell was mounted using MicroMount (Leica, Deer Park, IL, USA) and examined under a confocal microscope Zen Software (Zeiss LSM700) at 20× magnification.

### 4.14. Statistical Analysis

Statistical analyses were performed using GraphPad Prism version 8.0 for Mac (GraphPad Software, San Diego, CA, USA). The data were presented as mean ± standard deviation (SD). Differences between the groups were analyzed using a one-way analysis of variance (ANOVA), followed by Tukey’s post-hoc test. Comparisons with *p* < 0.05 were considered statistically significant.

## 5. Conclusions

This study showed that treatment with Fraction-7 of *X. muta* and LA presented no cytotoxicity, increased *SR-BI* transcriptional activity and mRNA expression of the *SR-BI* gene, and enhanced HDL uptake in HepG2 cells. The findings showed that Fraction-7 of *X. muta* and LA are safe and suitable as alternative controls, preventive agents, or adjuncts for hypercholesterolemia.

## Figures and Tables

**Table 1:** Cytotoxicity of diethyl ether fractions of *X. muta*.

Fractions of *X. muta*	Concentration of Fractions at 50% Cell Viability (μg/mL)	*SR-BI* Fold Change
Fraction 1	>100	0.62 ± 0.05
Fraction 2	55.30 ± 3.76	0.56 ± 0.02
Fraction 3	15.63 ± 1.66	-
Fraction 4	20.48 ± 1.65	-
Fraction 5	>100	0.51 ± 0.25
Fraction 6	>100	1.21 ± 0.36
Fraction 7	82.27 ± 1.26	1.64 ± 0.01

Data are presented as mean ± SD (*n* = 3).

**Table 2:** List of primers used to amplify *Luc-SR-BI* promoter sequence.

Gene	Primers	Sequence
*SR-BI*	Forward	5′-GATGATGGTCCCGATAGAGG-3′
*Luciferase*	Reverse	5′-GGTAGCTTCTTTTGCACGTTG-3′

**Table 3:** Primer for quantitative Rt-PCR analysis.

Gene	Primers	Sequence
*hSR-BI*	Forward	5′-CTG TGG GTG AGA TCA TGT GG-3′
	Reverse	5′-GTT CCA CTT GTC CAC GAG GT-3′
*h*-actin	Forward	5′-TCA CCC TGA AGT ACC CCA TC-3′
	Reverse	5′-CCA TCT CTT GCT CGA AGT CC-3′

## Data Availability

Data are available upon request.

## Supplementary Materials

The following supporting information can be downloaded at: https://www.mdpi.com/article/10.3390/md20120762/s1, Supplementary Table related to the spectra of the compounds presented in Fraction-7 of *X. muta* (GC-MS).
