# Supplementary material for: Xestospongia muta Fraction-7 and Linoleic Acid: Effects on SR-BI Gene Expression and HDL Cholesterol Uptake
Source: Mar Drugs. 2022 Dec 4;20(12):762. doi: 10.3390/md20120762 (PMC9784671; doi:10.3390/md20120762)
Supplement: Supplementary file 1 [file marinedrugs-20-00762-s001.zip › marinedrugs-1995094-supplementary.pdf]

Supplemental Table. Sterol and fatty acids composition in Fraction-7 of *X. muta* by GC-MS

No.	Retention Time (min)	Compound	Peak area (%)
1.	5.912	Methylamine	10.45
2.	6.080	Pyruvic acid	0.17
3.	7.021	2-Hexanol	0.15
4.	10.838	Lactic acid	0.43
5.	30.000	1-Tetradecanol	1.49
6.	30.189	Tridecanoic acid 4,8, 12-trimethyl	0.84
7.	30.078	Tetradecanoic acid, 12-methyl-, methyl ester	0.91
8.	31.501	Dodecanoic acid, 10 methyl-, methyl ester	1.64
9.	32.718	Hexadecanoic acid, methyl ester	0.64
10.	32.889	Pentadecanoic acid, 13-methyl-, methyl ester	0.64
11.	32.960	5-Isopropenyloxymethylene-3,3-dimethyl	0.08
12.	33.048	9-Hexadecenoic acid, methyl ester (Z)-	1.61
13.	33.152	Oleic acid, (Z)	0.24
14.	33.243	2-Piperidinone, N-{4-bromo-n-butyl}	0.08
15.	34.294	Methyl 9-methyltetradecanoate	0.59
16.	33.710	Dihydrophytol	2.12
17.	33.923	Pentadecanoic acid	0.25
18.	34.205	1-Hexadecanol	0.40
19.	34.294	Methyl 9-heptadecanoate	1.75
20.	34.379	Tetradecanoic acid 5,9,13 trimethyl	1.47
21.	34.440	Methyl 8, 10-dimethyl-hexadecanoate	0.64
22.	35.063	Tetrapentacontane 1,54-dibromo	0.20
23.	35.235	Methyl 9-eicosenoate	0.25
24.	35.543	Heptadecanoic acid, methyl ester	1.25
25.	35.991	Palmitic acid	2.17
26.	36.417	2-Hydrocyclohexane-1-carboxylic acid	0.25
27.	36.623	Arachidonic acid	0.24
28.	36.791	Methyl stearate	0.46
29.	36.865	9,12-Octadecadienoic acid, methyl ester	0.64
30.	36.994	6-Octadecenoic methyl ester	3.06
31.	37.109	11- Octadecenoic methyl ester	0.67
32.	37.290	15-Tetracosenoic acid	0.12
33.	37.812	Heptadecanoic acid	0.21
34.	37.933	Cycloheanone 2-(1 methyl-2nitroethyl)	0.13
35.	38.062	Beta-Citronellol	0.54
36.	38.239	Octadecanoic acid, 11-methyl-, methyl ester	3.22
37.	38.565	Methyl 8,10-dimethyl-hexadecanoate	0.26
38.	38.694	Octadecanoic acid, 17-methyl-, methyl ester	1.91
39.	38.860	Methyl 18-methylicosanoate	1.18
40.	39.093	9, 12-Octadecadienoic acid (Z,Z)	0.31
41.	39.197	Oleic acid, (Z)	1.74
42.	39.278	Oleic acid (Z)	0.13
43.	39.377	Octadecanoic acid, 17methyl-,methyl ester	0.34
44.	39.636	Stearic acid	1.07
45.	40.071	Butoxytriglycol	0.19
46.	40.092	Methyl 5, 13-docosadinoate	0.29
47.	40.421	5.alpha-dihydrotestosterone	0.35
48.	40.516	Methyl 18-methylnonadecanoate	2.10

49.	40.678	9-Hexadecenoic acid	1.47
50.	41.270	1-Hexacosene	0.22
51.	41.488	Hexadecanoic acid, 14-methyl-	0.69
52.	41.605	2,6,10-trimethylundecanoic acid	0.12
53	41.824	Methyl 14-methyl-eicosanoate	0.69
54.	42.435	Methyl 18-methyl-eicosanoate	4.92
55.	43.982	Butyl 6,9,12-hexadecatrienoate	0.84
56.	44.105	Methyl 14-methyl-eicosanoate	0.65
57.	45.649	5,8,11,14,17-Eicosapentanoic acid	0.21
58.	45.777	Cyclononasiloxane, octadecamethyl	0.24
